# Domestic Use of E-Cargo Bikes and Other E-Micromobility: Protocol for a Multi-Centre, Mixed Methods Study

**DOI:** 10.3390/ijerph21121690

**Published:** 2024-12-19

**Authors:** Ian Philips, Labib Azzouz, Alice de Séjournet, Jillian Anable, Frauke Behrendt, Sally Cairns, Noel Cass, Mary Darking, Clara Glachant, Eva Heinen, Nick Marks, Theresa Nelson, Christian Brand

**Affiliations:** 1 Institute for Transport Studies, University of Leeds, University Road, Leeds LS2 9JT, UK; 2 Environmental Change Institute, University of Oxford, South Parks Road, Oxford OX1 3QY, UK; 3 Industrial Engineering and Innovation Sciences, TU Eindhoven, 5612 AE Eindhoven, The Netherlands; 4 School of Humanities and Social Science, University of Brighton, Mithras House, Moulsecoomb BN2 4AT, UK; 5 ETH Zürich, Verkehrs- und Mobilitätsplanung, HIL F 31.3, Stefano-Franscini-Platz 5, 8093 Zürich, Switzerland

**Keywords:** e-micromobility, health, decarbonisation, physical activity, active mobility, study protocol

## Abstract

Physical inactivity is a leading risk factor for non-communicable diseases. Climate change is now regarded as the biggest threat to global public health. Electric micromobility (e-micromobility, including e-bikes, e-cargo bikes, and e-scooters) has the potential to simultaneously increase people’s overall physical activity while decreasing greenhouse gas emissions where it substitutes for motorised transport. The ELEVATE study aims to understand the impacts of e-micromobility, including identifying the people, places, and circumstances where they will be most beneficial in terms of improving people’s health while also reducing mobility-related energy demand and carbon emissions. A complex mixed methods design collected detailed quantitative and qualitative data from multiple UK cities. First, nationally representative (n = 2000), city-wide (n = 400 for each of the three cities; total = 1200), and targeted study area surveys (n = 996) collected data on travel behaviour, levels of physical activity, vehicle ownership, and use, as well as attitudes towards e-micromobility. Then, to provide insights on an understudied type of e-micromobility, 49 households were recruited to take part in e-cargo bike one-month trials. Self-reported data from the participants were validated with objective data-using methods such as GPS trackers and smartwatches’ recordings of routes and activities. CO_2_ impacts of e-micromobility use were also calculated. Participant interviews provided detailed information on preferences, expectations, experiences, barriers, and enablers of e-micromobility.

## 1. Introduction

E-micromobility (EMM) encompasses electrically assisted and lightweight two-, three-, or four-wheelers and includes vehicle types such as e-scooters, e-bikes, and e-cargo bikes [1]. E-micromobility is increasingly recognised in policy and academic debates as having the potential to deliver benefits in terms of transport decarbonisation and improved physical and mental health, two important aspects of public health. In this paper, we present the methodology of the ELEVATE project (https://environment.leeds.ac.uk/transport-social-political-sciences/dir-record/research-projects/1690/elevate, accessed on 15 December 2024). The main hypothesis of the ELEVATE project is the following: EMM has the potential to play a key role in improving physical and mental health while reducing mobility-related energy demand and carbon emissions. The project focuses on three EMM modes, e-bikes, e-cargo bikes, and e-scooters, which see increasing uptake. E-bikes and e-cargo bikes have legal basis for use in the UK, while e-scooters are currently limited only to some specific trial areas and to shared schemes. The Research Background section positions our study within e-micromobility research and illustrates research gaps in relation to our objectives.

The novelty and contribution of this study is not only that we add to the understanding of multiple e-micromobility modes across the UK—which has had lower uptake than several of its European neighbours—but also that we link these insights to in-depth fieldwork that is focused on more car-dependent suburban areas, which are less studied in terms of e-micromobility than city centres. By examining areas in Leeds, Brighton, and Oxford in the UK, the study encompasses a range of physical and socio-economic suburban contexts in provincial cities, which is critical for generating generalizable insights. Furthermore, we study e-cargo bikes, which are seen as novel in the UK, and we study their domestic use, which has received little attention compared to freight use. Methodologically, this study aims to offer a nuanced understanding of EMM’s impacts through a comprehensive and mixed methods approach, including stakeholder interviews, surveys, and household trials.

## 2. Background

The potential to contribute to carbon emissions reduction and increased physical activity are two important aspects of e-micromobility research and we focus on these aspects in this section. Other areas of micromobility research studied in relation to health include collisions/user safety [2,3], exposure to air pollution [4], and reduction in transport related social exclusion [5,6].

The evidence on the effects of e-micromobility (EMM) on physical activity (PA) and public health is complex. Health benefits depend on the type of EMM used as they require different levels of physical activity. The use of pedelec e-bikes (https://www.gov.uk/government/publications/electrically-assisted-pedal-cycles-eapcs/electrically-assisted-pedal-cycles-eapcs-in-great-britain-information-sheet, accessed 15 December 2024) , for instance, has been shown to be a form of active mobility, which provides lower intensity PA than a conventional bicycle, but people tend to travel further so that overall PA levels are similar [7,8,9,10]. E-scooters and skateboards require some pushing, walking, and standing, but their contribution to PA is lower than traditional forms of active travel, with e-scooters providing light-intensity physical activity [11]. However, they may open up new possibilities for multi-modal trips which do include an active travel element, a second important (public) health aspect. A third aspect refers to mode shift. When car use is replaced by active mobility [12], this can bring benefits by providing a sufficient level of physical activity to improve health and well-being [13], which is increasingly being recognised by public health agendas across the world [14,15,16]. In terms of well-being and mental health, sufficient physical activity has been shown to reduce symptoms of depression and anxiety, enhance mood, increase self-esteem, and improve overall cognitive function [17,18].

Climate change is now regarded as the biggest threat to global public health [19]. In the UK, car use reduction of at least 20% is essential to reduce transport carbon emissions [20]. The Climate Change Committee has stated a need to reduce car use, seeing EMM as part of the solution [21]. The picture around EMM and reducing mobility-related carbon emissions is complex. Within current strategies aimed at achieving net-zero emissions in the transportation sector, EMM has great potential for carbon reduction in terms of its ability to reduce car use [22,23,24,25], not only for short trips but in some cases for medium length journeys or 8–20 km [26], while combined with public transport, it has potential to also reduce car use for longer journeys [27,28,29]. However, where EMM replaces traditional active modes, carbon emissions can also slightly increase. Also, shared schemes can have carbon implications, e.g., around the short lifespan of vehicles and their re-distribution [30,31]. The environmental benefits of EMM go beyond reductions in carbon emissions to include a lower occupation of public space [32]. Despite these complexities, overall, compared to car ownership and use (both electric and conventional), EMM has significant benefits [33].

There are a number of research gaps in terms of gaining a greater understanding of EMM in our UK-based study in terms of physical activity, carbon emissions reduction, as well as other issues associated with these modes of transport. We highlight these in relation to our objectives below.

**Objective** **1.** *Assess current and possible future uptake of EMM. This includes assessing the use, ownership, and attitudes towards e-bikes, e-cargo bikes, and e-scooters, but also identifying the current landscape of policy and governance concerning EMM in the UK.*

The UK has a low uptake of active travel and lower uptake of EMM compared to a number of nearby EU countries [34]. A gap here is that relatively few studies focus on the UK, when arguably there is a greater need for understanding because it lags behind.

**Objective** **2.** *Understand barriers and enablers of EMM uptake and more specifically the uptake of e-cargo bikes in a suburban domestic use setting in provincial cities.*

EMM studies frequently focus on the largest cities (e.g., London in UK) that feature the most comprehensive e-micromobility offer [35], higher cycling rates, higher levels of investment in infrastructure, and e-cargo bikes being closer to mainstream unlike other areas of the UK [36]. A gap here is that provincial cities receive less research attention. Also, there is less research emphasis on suburban, peri-urban, and rural areas than on city centres in transport research and within EMM research [37,38].

The rationale for studying UK suburbs also includes the fact that they generally have high levels of car ownership which can contribute to high energy consumption. The justification of high consumption behaviours (and associated high carbon emissions) is not well understood [39], and targeting high-energy users may be needed to effect an equitable energy transition, especially since transport inequity can also have health impacts. The strong car dependency of many suburbs produces negative effects on many aspects of health [12,40]. This presents the research gap of studying suburbs and areas outside major cities. Also, for the specific case of e-cargo bikes, there is the strong literature on the observed and potential benefits for freight and logistics use [41]; however, there is a great deal less research on e-cargo bike use in a domestic context.

**Objective** **3.** *Understand the impact of EMM (particularly e-cargo bikes) ownership and use on physical and mental health as well as well-being.*

The WHO produces the Health Economic Assessment Tool, HEAT [42]. Whilst it provides outputs for e-bikes, it does not do so for e-cargo bikes or other EMM modes, partly due to there being only a small number of studies which investigate the physical activity levels associated with e-cargo bike use [43].

**Objective** **4.** *Establish the impact of EMM (particularly e-cargo bikes) ownership and use on energy consumption and lifecycle carbon emissions.*

There are few studies focused on domestic e-cargo bike use (see above) and to our knowledge, none specifically focused on the UK. This presents a research gap, including estimating the carbon and health implications arising from an empirical study of usage.

**Objective** **5.** *Provide insights into EMM implications for industry, policy, and end users.*

While various trials and pilot programmes have explored the potential of EMM in terms of health or reducing car use, few use multiple research methods. Whilst there have been some mixed methods studies, notably [44,45,46,47,48], these are in the minority. Mixed methods research can provide a wider range of insights relevant to a wider range of stakeholders than a single method study [49]. Using multiple mixed methods also provides an in-depth understanding of the state and potential of EMM in the UK to contribute to improved public health and decarbonisation. The combination of national, city, neighbourhood, and household level data collection that we employ is particularly rare.

Additionally, as existing research on e-cargo bikes largely focuses on freight, and geographically on city centres, studying suburbs and a domestic ownership/long-term lease context rather than share schemes also broadens the range of insights for policy makers, potential users, and other stake holders.

In summary, the research gaps identified are that EMM research is less developed where the focus is on the suburbs of provincial cities, while mixed-method approaches remain in the minority. The UK as an EMM laggard compared to some EU neighbours may benefit from research into EMM in a specific UK context. Further specific gaps regarding e-cargo bike are that there are few studies of domestic use.

## 3. Materials and Methods

To address the objectives of the ELEVATE project, a complex mixed methods research approach has been employed. This involves quantitative and qualitative data collection and analysis conducted at different levels. In this section, we provide an overview of the approach and then provide some additional detail about individual components.

First, stakeholder interviews were conducted to assess the current policy and governance landscape concerning EMM within the UK. This step was critical for identifying the roles and perspectives of key actors within the industry. This, along with an analysis of the literature, informed the design of the other data collection activities.

Second, quantitative methods, specifically structured surveys, were utilised to gather data on the existing ownership, usage patterns, and demand for EMM. This approach provided a broad-scale understanding of the current engagement with these transport modes.

We then adopted a mixed methods approach to explore the potential uptake of e-cargo bikes for suburban domestic use (not as part of a share scheme) through surveys and a trial intervention. Recognising that early adoption is likely skewed towards suburban geo-demographic segments, and that these groups live in more car dependent areas and have car dependent practices, this nuanced approach enabled an investigation into how e-cargo bikes could facilitate a modal shift towards reduced car use and carbon emissions and increased overall PA. This approach analyses not only individual behaviours but also the geographic, social, structural, and governance contexts that influence adoption patterns. The field trials conducted in Brighton, Leeds, and Oxford integrated various data collection methods including GPS tracking, physical activity monitoring, semi-structured interviews, travel diaries, and user surveys. This comprehensive data collection facilitated an in-depth analysis of the end-user perspective on using e-cargo bikes.

The ELEVATE study design builds on the protocols of similar studies in the field of micromobility, active travel, and associated health impacts [44,45,46,47] as well as empirical studies on e-bikes [48]. The study protocol has been approved by the University of Leeds’s Institutional Ethics Committee (Reference FREC 2023-0477-1198). Details of the University of Leeds ethics policy are provided at https://secretariat.leeds.ac.uk/research-ethics/protocols-and-policies/, accessed 15 December 2024.

### 3.1. Stakeholder Interviews

**Aim**: To obtain qualitative data and insights into the opportunities, barriers, and issues associated with EMM amongst policy stakeholders, transport planning practitioners, and those involved in the EMM industry.

**Data Collection**: Online and face-to-face semi-structured interviews with 11 stakeholder organisations. These included city, county, and parish councillors, sustainable mobility and active travel officers and planners, local and regional micromobility providers, bike shop managers, cycling instructors, and members of cycling and active travel charities, clubs, and NGOs. Interviews were tailored to each organisation.

We also had meetings with stakeholders and attended events and conferences organised for industry bodies and policy makers. Recruitment to the stakeholder interviews was carried out using the research team’s network of contacts. We also used snowballing to reach further contacts.

### 3.2. Nationally Representative Survey (NRS) and City Representative Surveys

**Aim**: To assess current awareness, ownership, and use of, attitudes towards, and potential adoption of EMM (RQ2),and the physical activity of respondents on a national scale (RQ3).

**Data Collection**: A nationally representative sample of 2000 English adults was obtained, with representation based on age, gender, region, ethnicity, and social grade. The survey was administered online by survey company Yougov and distributed by them to members of their panel. Data collection took place between 31 May and 18 July 2023. The survey focused on socio-demographic characteristics, existing travel behaviour and vehicle ownership, and perceptions and attitudes that may influence EMM awareness and adoption. Physical activity behaviour was measured by (1) a PA single item [50] and (2) the Global Physical Activity Questionnaire (GPAQ) with walking, cycling, and e-biking separated [51]. This survey was also run in three distinct UK cities (Brighton, Leeds, and Oxford) for which a representative sample was collected (n = 400 each). As part of the ethical approval process, we received confirmation from YouGov that their data management processes are GDPR compliant.

### 3.3. Intervention: Household E-Cargo Bike Trials

#### 3.3.1. Study Area Selection

Suburbs of provincial cities are more car-dependent than metropolitan urban centres. Therefore, three different suburb types were chosen in cities where the research teams are located (Leeds, Brighton, and Oxford). Key selection criteria were that they contained some neighbourhoods with high levels of car ownership (based on census data, as a proxy for car dependence) and property types with storage such as garages (based on census data combined with visual inspection of Google satellite and Streetview images). For practical reasons, the study areas had to be easily accessible to members of the research team. We also discussed study area selection with local stakeholders. Beyond this, we sought variety in terms of physical capability to substitute car use with e-cargo bike use [26], total household energy use [52], levels of deprivation, and accessibility to jobs, services, and activities. When examining census and other spatial data we used LSOA resolution data. LSOAs are Lower Layer Super Output Areas, which are UK census data dissemination units containing an average of approximately 700 households)

#### 3.3.2. Study Area Survey (SAS)

**Aims**: To carry out a baseline survey of the study area that is comparable to the national survey and reach potential trial participants.

**Data Collection**: Our survey was an abridged version of the NRS, designed to assess neighbourhood-specific variations in EMM perceptions and potential barriers to adoption. Additionally, this survey asked if respondents wished to take part in an e-cargo bike trial, as part of the trials’ recruitment. Data collection took place between 24th April and 30th September 2023 (n = 996). A follow-up survey was run after one year to record any changes. A prize draw was offered to respondents in the study area survey, and 3 GBP 100 vouchers were offered per city (Leeds, Brighton, Oxford).

**Recruitment**: To advertise the SAS, Meta (also known as Facebook) was the primary recruitment tool. We created business pages for each of the three locales to create targeted adverts and to be able to join local Facebook groups and advertise our survey within them. We used paid advertisements in each city [12 adverts reaching 24,210 potential participants] and posted in localised Facebook groups in each city [10 groups in Leeds, 8 groups in Oxford, 17 groups in Brighton; based on Facebook group followers, there were a total of 191,162 potential respondents]. We asked the groups to share a link and digital flier to our study area survey. Additional promotion was via contacting schools, placement of flyers on community noticeboards and contacting local stakeholders. We also anticipated some potential for snowballing—where a participant would encourage others to take part.

#### 3.3.3. Household Trial Recruitment and Implementation

**Figure 1:**
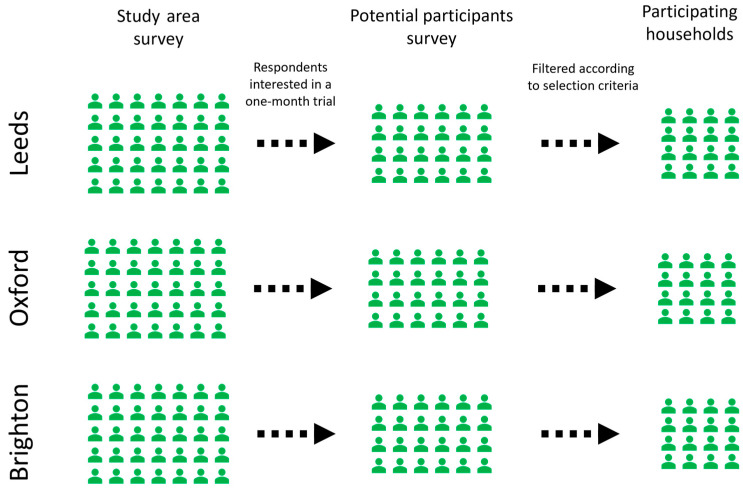
Recruitment of the trial participants.

Participants were recruited from the baseline survey of the study areas (Section 3.3.2), using a follow-up survey, then a consent discussion. This process is summarised in Figure 1.

##### Potential Participants Survey (PPS)

**Aim**: To recruit participants for e-cargo bike trial loans in the study areas.

**Recruitment**: Potential trial participants were initially identified from the SAS. They were sent this Potential participant survey (PPS) within a week of filling in the SAS with no obligation to become a trial participant.

**Data Collection**: PPS respondents were asked for details regarding storage, availability to take part, and further information about their intended use of the e-cargo bike.

##### Participant Selection and Consent

Participants were shortlisted following the PPS (see Figure 1). The selection criteria are as follows:

- Availability of both an e-cargo bike and the participant for a specific month/cohort: the focus was to give e-cargo bikes to households which would not abandon the bike during summer holidays due to travelling or other commitments;
- Availability of a safe and secure storage place, either inside the house or in a locked shed or garage;
- Expressed intended use for the bike: households who expressed an intention to use the bike frequently and for multiple purposes were preferred;
- Expressed intention to reduce and/or replace car journeys;
- Availability of preferred e-cargo bike type in Leeds, Oxford, and Brighton offered only one type of bike;
- Attempted diversity of the sample: the aim was to recruit households of different structures and socio-economic characteristics.

Then, the project information and consent forms were shared with participants prior to a discussion with a researcher, and the participants were given time to decide on participation.

##### E-Cargo Bike Loan

Participants were loaned the e-cargo bikes for one month for their household use, for free. The trials comprised four 1-month cohorts between June and mid-October 2023. Summer months are associated with higher levels of cycling due to more cycle-friendly weather [53]. The selected trial months covered both regular traffic months and the summer holiday period. Each city had 4 e-cargo bikes available for loan. Leeds secured a free loan of a fifth e-cargo bike for a short period.

Participants were encouraged to use the bikes as they needed or desired, and the researchers did not impose specific usage targets in terms of cycling distance, frequency, timing, or purpose. However, participants were expected to report on reasons or barriers that deterred them from using them plus arising technical issues or incidents.

The intervention was not simply the provision of a bike but contained several hard and soft measures. Participants were (1) supported in their use, including being given training, (2) provided with locks, (3) given helmets if they required them, and (4) given accessories such as child seats and rain covers to meet their needs. Some residents needed a ramp to enable storage up steps. Support included weekly contact where researchers discussed emerging issues and potential solutions, i.e., extra training or equipment, as early as possible. During the last two weeks, participants were invited to participate in a voluntary challenge: living as a car-free household and adjusting their travel behaviour and/or transport mode choice and decisions accordingly.

Researchers arranged for a bike handover session with each household to introduce the bike: its operation modes, techniques, components, and accessories. General cycling tips and knowledge were communicated, and participants’ queries were answered.

Each household had one mandatory training session, a condition of the project’s insurance and risk assessment. Follow-up sessions were also offered on a voluntary basis. Training was delivered by a city-based qualified National Standard cycling instructor. This was to boost households’ familiarity with the bike and its operation as early as possible and to encourage them to use the bike through addressing any pre-existing use fears and worries.

#### 3.3.4. Household Trial Data Collection

**Figure 2:**
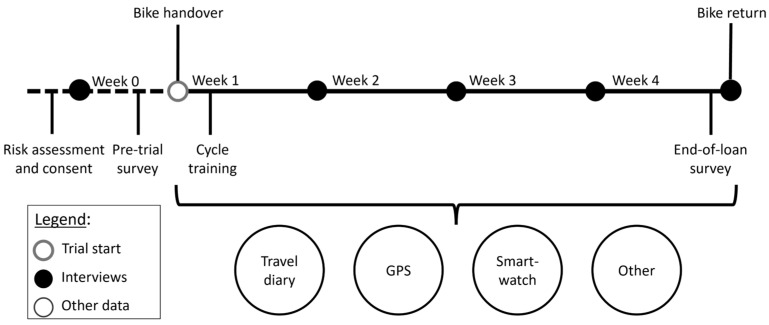
Trial timeline and collected data.

In total, the data collection ran in four cohorts with 4 e-cargo bikes available in each of the three cities. During their trials, the following data were collected (see Figure 2):

##### Pre-Loan and End-of-Loan Surveys

**Aim**: To evaluate the impact of EMM on trial participants’ attitudes towards e-cargo bikes but also to assess changes in physical activity.

**Data Collection**: Each participating household was asked to complete a pre-trial survey one week before the trial and an end-of-loan survey during the last week of the trial. For PA, GPAQ was administered for each wave.

##### Semi-Structured Participant Interviews

**Aim**: To obtain in-depth insights into participants’ experiences, expectations, hopes, concerns, and perceptions about e-cargo bike adoption and use, physical and mental health to develop an understanding of the enablers, and barriers to use. It also gave researchers weekly contact with participants to support them.

**Data Collection**: The interviews were conducted and recorded, as appropriate, face-to-face (with voice recordings) or online (recorded via Teams or Zoom). The interview schedule was to conduct an interview with each participant a week before the trial, and at the end of each week during the trial.

**A. The pre-trial interviews** established data to augment the PPS data, gathered details on existing travel habits and further explored motivations to participate. Baseline data were collected to allow for the comparison of expected versus actual usage and pre- and post-trial expectations. It also confirmed participations, after a pre-trial consent discussion took place.

**B. Weekly interviews** discussed e-cargo bike use. Crucially, non-use was also discussed, addressing the reasons and influences underlying this, obtaining vital data on the subjective and objective barriers to use. Participants were also asked to describe some of the non-cargo bike journeys they made. This also contributed to building a picture of the practices involved in ‘e-cargo bike citizenship’, and to look at the physical and mental health impacts that these practices entrained.

**C. End-of-loan interviews** delved into how—if at all—the bike changed participants’ and/or their households’ travel habits, mode choice, and whether it was incorporated in their day-to-day travel decision making. Further, the bike’s impact on physical activity patterns and mental well-being was examined. Logistical problems with operating the bike covering storage, security, parking around town, ease of use, cycling infrastructure, and the cities’ readiness to accommodate e-cargo bikes, and what the participants felt would improve this, were also discussed. Finally, the final weekly interview explored participants’ willingness to use/buy an e-cargo bike in the future and whether they could see it replacing/reducing their car use.

The interview data were analysed with codes developed by the project team. This combined deductive coding, drawing on our interview schedule, and inductive coding, via early co-coding sessions, all outlined in a codebook. Four researchers engaged in the collaborative coding of the interview transcripts, using NVivo 14 cloud collaboration software. While three of these researchers participated in data collection, the fourth was uninvolved, to mitigate bias. We employed a qualitative approach to intercoder reliability, involving multiple rounds of test coding on the same interview transcript and subsequent group discussions on divergences and overlaps. We then collectively established coding strategies, e.g., for text segmentation, co-coding, and a shared understanding of codes. The dataset could be queried according to the intersection of relevant codes and classifications, to illustrate and qualitatively test hypotheses against the data segments outputted by such queries.

##### Travel Diaries

**Aim**: To collect data on the use of the e-cargo bikes in the trials, and to record car use before, during, and after trial participation.

**Data Collection**: Paper travel diaries were used to record e-cargo bike usage. The data fields included journey dates, times, duration, number of passengers, weather, purpose, level of need, and whether the journey substituted another mode of travel and reasons for doing so. Odometer (mileage) readings were taken for all participant cars before, during, and after the trial to record any change in vehicle usage. The diaries were collected and digitised.

##### Smart Fitness Watches

**Aim**: To collect objective data indicators of the exercise intensity (indirect measures using heart rate) observed when participants used e-cargo bikes.

**Data Collection**: The trial participants were equipped (voluntary) with a smart mobile phone and a smartwatch—FitBit Sense—to monitor their physical activity [54,55]. These devices captured heart rate and other data such as location and route data. Those who already had a smartwatch were invited to download and share their exercise data.

##### GPS Tracking

**Aim**: To capture objective data on the use of the e-cargo bikes in the trials, relating to journey origins and destinations, routes, distances, durations, and each bike’s live location. The trackers aided management of the fleet and boosted its security. They also helped in matching/triangulating the recorded journeys with those documented on the paper travel diaries.

**Data Collection**: E-bike GPS tracking units—BikeTrax by PowUnity—were installed and activated on the bikes before the trials. The trackers automatically recorded almost all the participants’ trips without the need for intervention or action from the participants.

##### Other

**Aim**: To gather other forms of evidence about participant experience during the e-cargo bike trials, and to gain reflections from the wider community about EMM use.

**Data collection**: Participants, on voluntary basis, sent photos, videos, and reflections via emails and WhatsApp. Researchers also recorded their own observations.

Table 1 provides a summary of the data collected.

## 4. Discussion

The ELEVATE project addresses a crucial gap in the literature by focusing on the potential of e-micromobility (EMM) to enhance public health and reduce carbon emissions specifically by domestic users in suburban and peri-urban areas. This study aims to offer a nuanced understanding of EMM’s impacts through a comprehensive and mixed methods approach, including stakeholder interviews, surveys, and household trials.

ELEVATE’s design is innovative, targeting suburban regions with high car dependency. This focus diverges from many existing studies that primarily investigate core urban settings, thereby broadening the understanding of EMM’s applicability and benefits. By examining areas in Leeds, Brighton, and Oxford in the UK, the study encompasses a range of physical and socio-economic suburban contexts, which is critical for generating generalizable insights.

The mixed methods approach integrates quantitative data from surveys, GPS tracking, and health monitoring with detailed qualitative insights from interviews and travel diaries. This combination is robust [49], allowing for a detailed examination of both measurable outcomes and personal experiences. The use of smart fitness watches and GPS technology provides precise data on physical activity and travel behaviour, enhancing the reliability of the findings.

ELEVATE is poised to contribute significantly to the existing literature by assessing EMM uptake, identifying barriers and enablers, evaluating health impacts, and measuring environmental impacts. The study will identify current usage patterns and future adoption potential, filling a gap in understanding EMM’s appeal in non-urban areas. By exploring factors such as cost, convenience, safety, attitudes, and social norms, the research will provide a comprehensive view of what influences EMM adoption and use, extending beyond the urban-centric insights prevalent in current studies. The project will measure EMM’s effects on physical activity and mental well-being, contributing to the public health literature that often overlooks the transportation–health nexus in suburban contexts. Additionally, by analysing changes in car ownership and use and potential corresponding decreases in carbon emissions, ELEVATE will offer empirical data to support EMM’s role in achieving environmental sustainability goals. The findings will provide actionable insights for policy makers and stakeholders, guiding the development of strategies to promote EMM in similar settings.

While ELEVATE’s approach is comprehensive, the reliance on self-reported data and the potential for selection bias in household trials must be acknowledged. Additionally, the study’s suburban focus, while novel, may limit the applicability of findings to more densely populated urban areas. However, the project’s rigorous methodology and targeted scope are strengths that address significant gaps in the current literature, particularly regarding the intersection of transportation, public health, and environmental sustainability in suburban contexts.

## 5. Conclusions

In conclusion, the novelty of this ELEVATE study is that it not only considers national patterns of EMM adoption, but that it does so in conjunction with city, neighbourhood, and household level data, while it also focuses on suburbs and peri-urban areas in medium-sized cities, which are generally more car-dependent, than the previously studied urban centres. Through its surveys, it allows a comparison of uptake use and perceptions of several e-micromobility modes at national level and combines this with the research trial which is novel in that it is the only mixed method study we are aware of focusing on domestic e-cargo bike use at multiple sites in the UK, which addresses the research gap of understanding the potential of e-micromobility (EMM) to enhance public health and reduce carbon emissions in those locations. Its methodological rigour and focus on under-researched settings position it as a critical study that can inform future transportation policies and public health initiatives.

Driven by its protocol, ELEVATE is strongly set up to provide valuable insights into the broader applicability of EMM beyond urban centres. This will not simply inform the promotion of wider uptake of e-micromobility, but also add to the understanding of where exactly increased e-micromobility use can contribute to positive health outcomes, transport decarbonisation, and energy demand reduction.

## Figures and Tables

**Table 1:** Summary of the data collected.

Data Type	Number of Respondents or Participants
Stakeholder interviews	11
Nationally representative survey	2000
City representative surveys	400 in Leeds, 400 in Oxford, 400 in Brighton; total = 1200
Study area survey	996
Potential participant survey	155
E-cargo bike loan	49
Semi-structured participant interviews	
Pre-trial interview	1 per participant, total = 49
Weekly interviews	3 per participant, total = 147
End-of-trial interviews	1 per participant, total = 49
Travel diaries	1 per participant, total = 49
Smartwatches’ data	18 participants recorded 227 trips between them
GPS tracking	46 participants used bikes fitted with GPS trackers

## Data Availability

Data will be archived, once anonymised, at https://archive.researchdata.leeds.ac.uk/.
